# Serodiagnosis of amoebic abscess: a retrospective diagnostic accuracy study of kits marketed in Europe

**DOI:** 10.1128/jcm.00179-25

**Published:** 2025-10-21

**Authors:** E. Prétot, M-P. Brenier-Pinchart, P. Tirard-Collet, F. Gabriel, F. Touafek, A. Marteau, L. Delcey, C. Amiot, D. Dupont, H. Fricker-Hidalgo, H. Sokol, A. Moreno-Sabater, F. Grenouillet

**Affiliations:** 1 Université Marie et Louis Pasteur, CHU Besançon, CNRS, Chrono-Environnement (UMR-6249) 27000, Besançon, France; 2 Université Grenoble Alpes, CHU Grenoble 36724 , Grenoble, France; 3 Université Lyon I, Hospices Civils de Lyon, Hôpital de la Croix-Rousse, Lyon, France; 4 Centre Hospitalier Universitaire de Bordeaux 728253 , Bordeaux, Nouvelle-Aquitaine, France; 5 Assistance Publique–Hôpitaux de Paris (AP-HP) - La Pitié-Salpêtrière – Charles-Foix 26930 , Paris, France; 6 Université Sorbonne Paris Nord, AP-HP – Avicenne 167549 , Bobigny, France; 7 CHU Besançon 55049 , Besançon, France; 8 Sorbonne Université, INSERM, Centre de Recherche Saint-Antoine (CRSA), AP-HP, Hôpital Saint-Antoine 27063, Paris, France; 9 INRAe, Micalis & AgroParisTech, Jouy-en-Josas, France; 10 Paris Centre for Microbiome Medicine (PaCeMM) FHU, Paris, France; 11 Centre d'Immunologie et des Maladies Infectieuses, Sorbonne Université, INSERM, AP-HP, Hôpital Saint-Antoine 27063, Paris, France; 12 PaCEMM FHU 669741, Paris, France; Mayo Clinic Minnesota, Rochester, Minnesota, USA

**Keywords:** amoebiasis, serology, ELISA, latex agglutination, indirect hemagglutination

## Abstract

Amoebiasis is a cosmopolitan parasitic disease caused by *Entamoeba histolytica*. The most lethal form is extra-intestinal amoebiasis, mainly manifesting as liver abscesses. Diagnosis is based on clinical, radiological, and biological tests. However, there are currently few serology reagents still available on the European market for *in vitro* diagnosis, and comparative studies of existing reagents are required. We evaluated the performances of the four currently available reagents, either as standalone tests or in combination. Two enzyme-linked immunosorbent assays (ELISAs) for the detection of *E. histolytica* IgG were assessed: one manufactured by Bordier and the other by NovaTec. Additionally, an indirect hemagglutination technique, ELI.H.A *Amoeba*, and a latex particle agglutination technique, ELITex Bicolor *Amoeba*, both produced by ELITech Microbio, were evaluated. A total of 442 serum samples were selected from a shared centralized biobank of seven university hospitals in France. The samples included 79 from patients with amoebic abscess, 13 with amoebic colitis, and 350 from healthy donors and patients with parasitic and non-parasitic diseases, especially liver disease and immune dysfunction. The sensitivity of the four kits ranged from 87.3% to 97.5%, while their specificity ranged from 78.3% to 98.6%. Bordier ELISA demonstrated the highest sensitivity, while ELITex Bicolor *Amoeba* latex reagent exhibited the highest specificity. The combination of Bordier ELISA and/or ELI.H.A *Amoeba* for screening, combined with ELITex Bicolor *Amoeba* for confirmation of positive screening results, yielded the most optimal performance. This study highlights the benefits of large comparative studies of commercial assays to guide clinical microbiologists in their choices.

**IMPORTANCE:**

Amoebiasis caused by *Entamoeba histolytica* is common in countries with low socio-economic levels. The most lethal form is extraintestinal amoebiasis, mainly liver abscesses, which require rapid and accurate diagnosis. Diagnosis is based on clinical, radiological, and, most importantly, serological tests. Implementing a diagnostic strategy requires both knowledge of how diagnostic tests compare with each other and thorough cross-validation. To help the clinical microbiologist choose a serological reagent, we evaluated the performance of four serological reagents currently available on the European market using a large biobank of sera from seven French university hospitals. In addition to patients with amoebic abscess and healthy donors, our study included many samples from patients with other parasitic and non-parasitic pathologies (liver diseases, immune dysfunctions) in order to study particularly non-specific reactivities.

## INTRODUCTION

Amoebiasis represents a significant global health concern, with a high mortality rate. Of the 500 million individuals infected with *Entamoeba* spp. (1), 10% are infected with *Entamoeba histolytica* (*Eh*)*,* the only species known to be pathogenic to humans. Invasive forms of amoebiasis, including amoebic liver abscess, are responsible for 50 000 to 70,000 deaths per year (2, 3).

In non-endemic regions, travelers and migrants are the main populations affected. The diagnosis of amoebiasis presents a significant challenge. Digestive forms could be primarily misdiagnosed as inflammatory bowel diseases, and distinguishing amoebic liver abscess from pyogenic liver abscess is difficult. This diagnosis challenge may lead to delayed and/or inadequate management, such as longer diagnostic delay or prescription of inappropriate antibiotics. Serology is primarily employed in the diagnosis of invasive *Eh* forms. Although serology can also provide information for diagnosing *Eh* colitis, the gold standard techniques for diagnosis in these cases remain parasitological and/or molecular examination of stool samples (2–4).

In many countries, there is a growing trend towards standardizing diagnostic reagents used in hospital laboratories. This shift has resulted in the abandonment of in-house techniques in favor of commercial assays labeled for *in vitro* diagnosis. With stricter European regulations regarding the European Conformity marking (5), the distribution of some diagnostic assays was discontinued by manufacturers, i.e., immunofluorescence assays (*Amoeba* Spot IF, bioMérieux) and Ridascreen enzyme-linked immunosorbent assay (ELISA) (R-Biopharm). As a result, the number of commercial assays for amoebic abscess has been drastically reduced. Re-evaluation of the available reagents is therefore required to implement adequate diagnostic strategies depending on laboratory needs.

Implementing a diagnostic strategy requires both knowledge of how diagnostic tests compare with each other and thorough cross-validation. With this in mind, we carried out a multicenter retrospective study to compare four available serology reagents for diagnosis of amoebic abscess, using a centralized biobank of 442 serum samples from seven university hospitals in France.

## MATERIALS AND METHODS

### Study design and human samples

This study involved seven parasitology-mycology laboratories in France. Serum samples were collected by each laboratory within the last 10 years and had not undergone repeated freeze-thaw cycles. Samples were centralized in the biobank of the Besançon serology laboratory (SINPAF biobank, authorization AC2023-946) and stored at −20°C until testing. To ensure integrity, samples were excluded if the results were not concordant with the initial results obtained using the same technique.

In order to assess sensitivity or specificity of 95% with an α-risk of 0.05 and a marginal error of 0.05, the required total sample size was 73 (6). Serum samples (one serum sample selected per patient) were collected at the time of diagnosis or during follow-up of liver disease, echinococcosis (up to 1 year), and other parasitic diseases (up to 3 months). Patients were diagnosed with *Eh* abscess (*Eh*A), *Eh* colitis (*Eh*C), non-*Eh* disease (N*Eh*D), such as parasitic disease (helminthiasis or protozoan disease), liver disease or immune dysfunction, or isolated positive *Eh* routine serology. Healthy donors (HDs) of blood or fecal matter were also included. The diagnostic criteria defining the *Eh*A group were a combination of positive amoebic routine serology, consistent epidemiology, imaging, and treatment with metronidazole resulting in therapeutic success. If available, positive molecular biology was also an argument. The subgroup defined by an "isolated positive *Eh* routine serology" included patients with positive amoebic serology for one or more *Eh* techniques, as well as an acute episode that was clinically consistent with an amoebic abscess. However, the diagnosis of amoebiasis was rejected by a multidisciplinary team, and the patients were not treated for this pathology. Other sample selection criteria are described in Table S1.

For patients with an immune dysfunction, serum samples with at least one autoantibody titer greater than 2N (among rheumatoid factors and/or antinuclear antibodies and/or anti-citrullinated peptide antibodies) were selected (N: threshold value for positivity of each antibody respectively defined in the Besançon immunology laboratory). However, for most samples, the levels of autoantibodies were greater than 5N for at least one parameter.

Patient clinical data, including age, sex, date of diagnosis and sample collection, treatment, and outcome, were obtained from medical records.

### Serological assays

Four commercial kits were evaluated: ELITex Bicolor *Amoeba* (latex agglutination (LA)) and ELI.H.A *Amoeba* (indirect hemagglutination [IHA]) manufactured by ELITech Microbio (Signes, France), and two *Eh* IgG ELISA kits, one from Bordier Affinity Products (Crissier, Switzerland) and the second from NovaTec Immundiagnostica GmbH (Dietzenbach, Germany).

The same operators used the commercial kits blindly at a single center, over a period of 2 months. Tests requiring macroscopic reading (IHA and LA) were read blindly in duplicate. Assays were conducted following manufacturers’ instructions, and results were interpreted using manufacturer cut-off values (Table S2). Equivocal results were considered negative. Concerning ELITex Bicolor *Amoeba,* the test was performed semi-quantitatively, allowing for the calculation of a titer. Briefly, a first assessment was performed at a 1:5 dilution. When agglutination was observed, the result was considered positive, and serial 1:2 dilutions were carried out up to 1:40 dilution. A second dilution (1:10) was also performed for samples that initially tested negative (1:5 dilution) by this test but having yielded a positive result in at least one other technique assessed in this study, so as to discard a potential prozone effect.

### Statistical analysis

The test parameters were determined using 2 × 2 contingency tables, which allowed for the calculation of sensitivity, specificity, likelihood ratios (LR), area under the curve (AUC), and accuracy. The gold standard was an established diagnosis of amoebic abscess, determined by a combination of positive serology, consistent epidemiology, imaging, and successful treatment outcomes (Table S1). Results were analyzed using receiving operating characteristic (ROC) curves and the Youden index to determine the most appropriate threshold(s) for each ELISA test. The Youden index enables the determination of the optimal combination of sensitivity and specificity for quantitative techniques. The new index established using the Youden index has been used to assess the performance of new techniques and optimize the thresholds of the two quantitative techniques.

Serodiagnostic strategies combining two techniques were also evaluated. Negative results obtained with the first sensitive technique were considered as the final result and were not confirmed, whereas positive results with the first technique were confirmed (or refuted) using a second, more specific technique, which was considered as the final result. Performance was therefore calculated according to these final results from the combination of the two techniques.

Serum samples from patients in the *Eh*C group were excluded from the performance analysis, as the selected kits are not indicated for diagnosis of amoebic colitis.

The data were subjected to a Wilcoxon-Mann-Whitney test for comparison. A *P*-value of less than 0.05 was considered statistically significant. Generation of figures, graphs, and curves was conducted using R Statistical Software version 4.3.1 (R Core Team 2021, Boston, MA, USA), GraphPad Prism version 8.0.1 (GraphPad Software, Boston, MA, USA) and Microsoft Excel version 2407 (Microsoft, Redmond, WA, USA).

## RESULTS

### Population characteristics

The study population included 442 patients. Demographic and diagnostic data are presented in Table S3. Patients included in the *Eh*A group (*n* = 79) had amoebic liver abscess, except one patient who had cecal abscess. Molecular detection of *Eh* DNA in pus aspirates confirmed the diagnosis in 31 out of 34 patients with *Eh* amoebiasis (*Eh*A group), for whom data were available (91%). The male-to-female ratio was 5.6, and the median age was 41 (range 2–74) (Table S3).

In order to analyze the kit performances, patients with other parasitic diseases, including eight helminthiases (*n* = 72) and six protozoan diseases (*n* = 62), or immune dysfunctions (*n* = 60), liver diseases (*n* = 29), or non-relevant isolated positive serology (*n* = 29) were included in this study (Table S1). Molecular biology ruled out a diagnosis of amoebic liver abscess in six out of the 29 patients of this last subgroup; the results were negative. Donors of blood (*n* = 80) and fecal matter (*n* = 18) were also included as the healthy population.

### Performances of four commercial tests for *Eh* tissular abscess diagnosis

Sensitivity of the four kits ranged from 87.3% to 97.5% (Table 1). Bordier ELISA was the most sensitive technique, with only two serum samples yielding false-negative results. ELI.H.A *Amoeba* showed a sensitivity value comparable with that obtained using Bordier ELISA, with 82% of *Eh*A serum samples exhibiting positivity with a titer ≥1,280 (Fig. S4C). ELITex Bicolor *Amoeba* demonstrated relatively lower sensitivity (89.9%), yielding eight false-negative results (8/79, 10.1%) (Table 1). Among the *Eh*A group, 41 out of 79 presented a titer higher than 40, and only 3 out of 79 sera yielded a titer of 5, which represents the lowest positive dilution (Fig. S4A). No evidence of a prozone effect was observed with the LA method.

**TABLE 1:** Serological results for 429 serum samples obtained using the four commercial kits for the serological diagnosis of *E. histolytica* abscess, and analytical performances with the manufacturer’s threshold^*a*^

	No. tested	Bordier ELISA		NovaTec ELISA		ELI.H.A *Amoeba*		ELITex Bicolor *Amoeba*	
		Pos	Neg	Pos	Neg	Pos	Neg	Pos	Neg
*Eh*A group (disease)	79	77	2	69	10	75	4	71	8
N*Eh*D + HD groups (no disease)	350	54	296	76	274	7	343	5	345
Se (%)	429	97.5 (97.4–98.8)		87.3 (87.3–88.6)		94.9 (94.9–96.2)		89.9 (89.8–91.1)	
Sp (%)		84.6 (84.5–85.8)		78.3 (78.3–79.5)		98.0 (98.0–99.3)		98.6 (98.5–99.9)	
AUC		0.984 (0.972–0.997)		0.905 (0.857–0.953)		0.983 (0.963–1.003)		0.948 (0.914–0.982)	
Accuracy (%)		86.9 (86.9–88.2)		80.0 (79.9–81.2)		97.4 (97.4–98.7)		97.0 (96.9–98.3)	
LR^+^		6.32 (6.31–6.65)		4.02 (4.02–4.05)		47.47 (47.38–47.61)		62.91 (62.50–63.09)	
LR^−^		0.03 (0.03–0.04)		0.16 (0.16–0.17)		0.05 (0.05–0.06)		0.10 (0.10–0.11)	

^*a*^ *Eh*A, *E. histolytica* abscesses; N*Eh*D, non-*E. histolytica *diseases; HD, healthy donor; Se, sensitivity; Sp, specificity; AUC, area under the curve; LR^+^, positive likelihood ratio, LR^-^, negative likelihood ratio; (X-X) 95% confidence interval; Pos, positive; Neg, negative.

**Fig 1:**
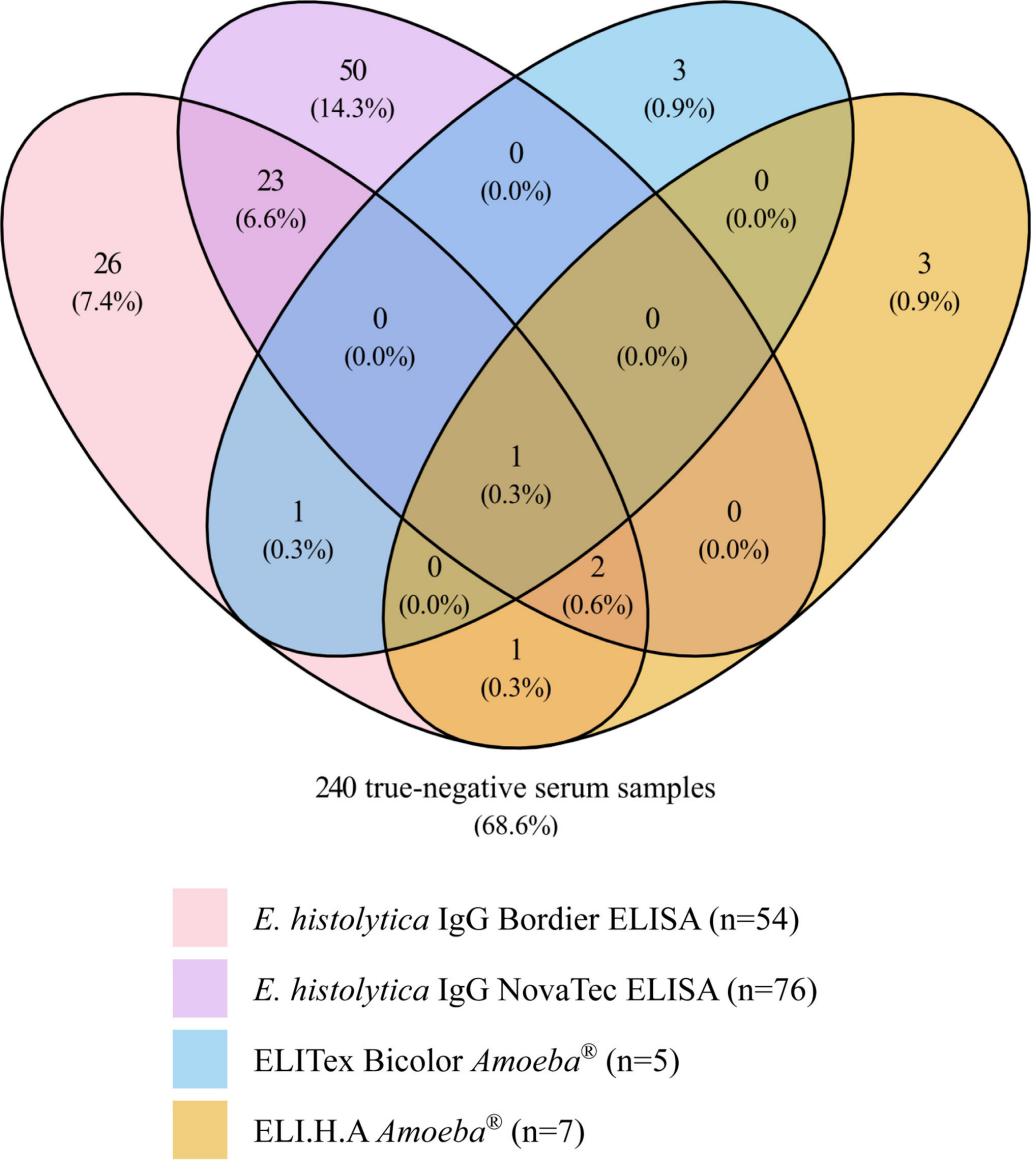
Venn diagram of false-positive results by technique. Distribution of false-positive results versus true-negative results according to the techniques among patients without *E. histolytica* disease, i.e., non-*E*. *histolytica* diseases and HDs (*n* = 350; false-positive results for at least one technique *n* = 110/350, 31.4%; and true-negative results *n* = 240, 68.6%).

ELITex Bicolor *Amoeba* demonstrated the highest specificity (98.6%), with only five false-positive results (Table 1; Fig. 1). This method offered the highest increase in post-test probability of the diagnosis of *Eh* abscess (LR^+^). Specificity of the ELI.H.A *Amoeba* test is close to that of the ELITex Bicolor *Amoeba* test (98.0%). The negative LR (LR^-^) of ELI.H.A *Amoeba* was less than 0.1 (Table 1), indicating a high degree of diagnostic benefit associated with a negative test result (i.e., post-test probability to exclude *Eh*A); the increase in post-test probability LR^+^ was also high. The specificity of the Bordier ELISA was lower than those described above. False-positive results were primarily observed in serum samples from patients with a single isolated positive serology but an excluded diagnosis of amoebiasis (14/54, 26%) (Table S5). A total of 76 false-positive serum samples were observed with NovaTec ELISA, leading to the lower specificity (Table 1).

Moreover, many false-positive results were described with one or both ELISA methods (90%, 99/110), whereas very few false-positive results were observed with the LA and IHA methods (Fig. 1). Of the 110 control serum samples (N*Eh*D and HD groups, *n* = 350) that yielded a false positive result, serum samples belonging to the liver disease or immune dysfunction disease groups had the highest number of false positives, regardless of the method employed (Table S5). In addition, false-positive results were also identified in patients diagnosed with filariasis, echinococcosis, leishmaniasis, and strongyloidiasis when analyzed by ELISA methods. Considering equivocal results as positive increases sensitivity and decreases specificity. The results of the two relevant techniques are provided in Table S6.

### Threshold adjustment

**Fig 2:**
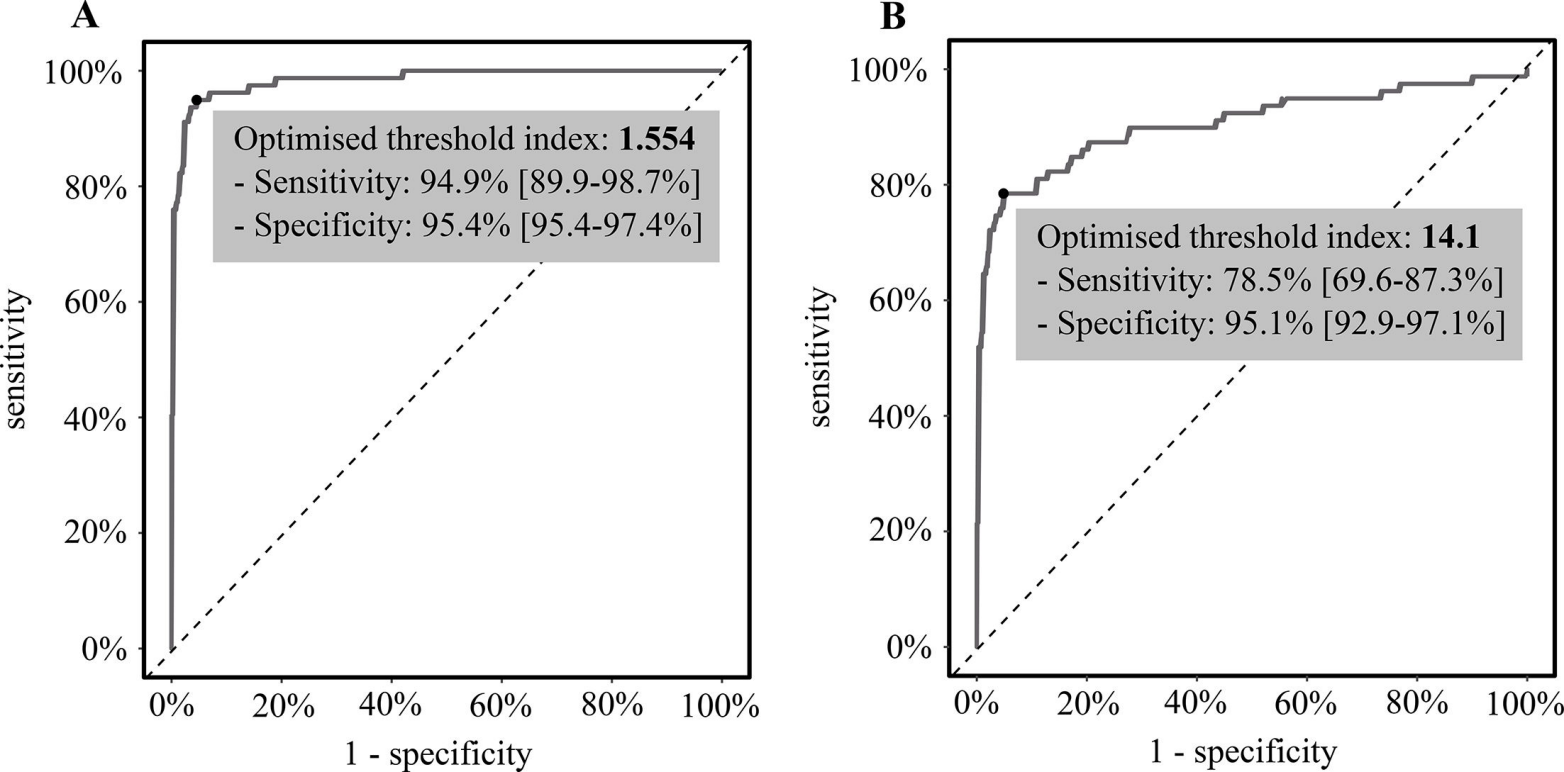
ROC curves for quantitative techniques. (**A**) *E. histolytica* IgG Bordier ELISA (*n* = 429), reference: diagnosis of amoebic abscess; (**B**) *E. histolytica* IgG NovaTec ELISA (*n* = 429), reference: diagnosis of amoebic abscess.

In order to improve ELISA assay performances, ROC curves were designed to identify the optimal threshold (Fig. 2A and B). For Bordier ELISA, the optimal Youden index was 0.9, obtained with an ELISA threshold index of 1.554 (Fig. 2A). In contrast, for NovaTec ELISA, the optimal Youden index was lower at 0.74, corresponding to an ELISA threshold of 14.1 NovaTec Units (NTUs) (Fig. 2B).

**Fig 3:**
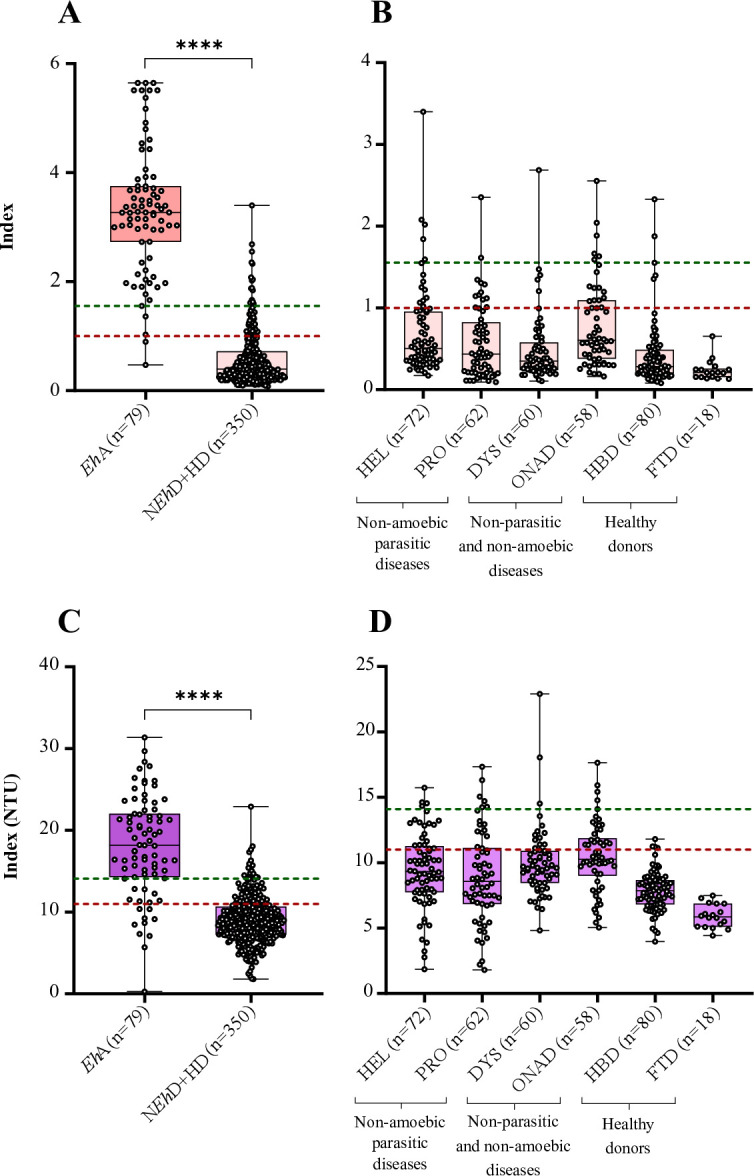
Representation of index values. (**A, B**) By group for *E. histolytica* IgG Bordier ELISA, according to manufacturer’s threshold (red line = 1) and optimized threshold index (green line = 1.554) in the total cohort (*n* = 429) (**A**) or focused on non-*Eh* (*n* = 252) and HD (*n* = 98) subgroups (*n* = 350) (**B**). (**C-D**) By group for *E. histolytica* IgG NovaTec ELISA, according to manufacturer’s threshold (red line = 11) and optimized threshold index (green line = 14.1) in the total cohort (*n* = 429) (**C**) or focused on non-*Eh* (*n* = 252) and HD (*n* = 98) groups (*n* = 350) (**D**). *Eh*A, *E. histolytica* abscesses; N*Eh*D, non-*E*. *histolytica* diseases; HD, healthy donor; HEL, helminthiases; PRO, protozoan diseases; DYS, immune dysfunctions; ONAD, others non-amoebic diseases (liver diseases and isolated positive serology); HBD, healthy blood donor; FTD, fecal transplant donors. ns, non-significant, ****: significant difference, <10^−4^.

The newly established thresholds were subsequently applied to the various populations evaluated in this study (Fig. 3). The new threshold allowed for the reduction of false-positive values without an increase in false-negative values for both methods. Table 2 presents a comparison of the performance of the ELISA techniques with the current manufacturer’s threshold and the optimized threshold. These optimizations increased the specificity of each ELISA to 95.4% and 95.1% for Bordier and NovaTec ELISAs, respectively, with a slight decrease in sensitivity for Bordier ELISA (94.9%) but a greater decrease for NovaTec ELISA (78.5%).

**TABLE 2:** Comparison of ELISA technique performances using optimized thresholds^*a*^

Commercial method	Threshold	Sensitivity (%)	Specificity (%)	Accuracy (%)	LR^+^	LR^−^
*E. histolytica* IgG Bordier ELISA	1 (manufacturer)	97.5 (97.4–98.8)	84.6 (84.5–85.8)	86.9 (86.9–88.2)	6.32 (6.31–6.65)	0.03 (0.03–0.04)
	1.554 (optimized)	94.9 (94.9–96.2)	95.4 (95.4–96.7)	95.3 (95.3–96.6)	20.77 (20.74–20.84)	0.05 (0.05–0.06)
*E. histolytica* IgG NovaTec ELISA	11 (manufacturer)	87.3 (87.3–88.6)	78.3 (78.3–79.5)	80.0 (79.9–81.2)	4.02 (4.02–4.05)	0.16 (0.16–0.17)
	14.1 (optimized)	78.5 (78.5–79.7)	95.1 (95.1–96.4)	92.1 (92.0–93.3)	16.16 (16.13–16.22)	0.23 (0.23–0.24)

^*a*^ LR^+^, positive likelihood ratio; LR^−^, negative likelihood ratio; (x-x), 95% confidence interval.

### Evaluation of serodiagnostic strategies

Strategies combining a sensitive first-line screening test with a confirmatory test for positive first-line results were evaluated. The combination of two screening commercial kits (Bordier ELISA and IHA) increased sensitivity to 97.5%. The combination of the most sensitive techniques (Bordier ELISA and/or IHA) confirmed by the LA technique provided specificity of 99.7%, with the highest post-test probability.

### *Eh* colitis

Eight out of the 13 samples from patients with amoebic colitis yielded positive results with all techniques employed, while three samples remained negative with all techniques (Table S7). In cases where serological tests yielded positive results, antibody levels were notably elevated. The median index for Bordier ELISA was 2.06, while NovaTec ELISA yielded a value of 16.73 NTU. Additionally, the median titers for IHA and LA were 5,120 and 20, respectively.

## DISCUSSION

The results of this multicenter retrospective accuracy study demonstrated that Bordier ELISA and ELI.H.A *Amoeba* exhibited the highest sensitivity for *Eh* serological diagnosis. The ELITex *Amoeba* LA kit had the highest specificity value, demonstrating a low incidence of false positives in comparison to the ELISA kits. When the LA method is combined with one of the two sensitive techniques, the proportion of false-positive results is improved, particularly those observed with ELISA techniques. Furthermore, the extensive cohort of serum samples used in this study made it possible to establish improved ELISA thresholds and to reduce false-positive results.

As previously reported (7), Bordier ELISA is a sensitive technique for the diagnosis of *Eh*A. However, our study revealed lower specificity than previously reported (84.6% versus 94.0%), probably due to the high diversity of sera included in our cohort, such as patients with liver disease or immune disorders. Concerning the ELI.H.A *Amoeba* technique, previously marketed as IHA Amibiase (Fumouze Diagnostics), our study obtained similar performances in terms of sensitivity and specificity to those previously reported (8–10). ELITex *Amoeba*, formerly marketed as Bichro-Latex *Amibe* test (Fumouze Diagnostics), has been described as a highly specific technique for *Eh* serological diagnosis (95%–99.5%) and with a sensitivity value comparable to the ELISA and hemagglutination methods (90.7%–97.8%) (1, 8–10). In contrast, our results revealed that the LA method was less sensitive in this study for the diagnosis of *Eh*A than Bordier ELISA and ELI.H.A *Amoeba*. NovaTec ELISA demonstrated suboptimal sensitivity and specificity, as previously reported in the study of Larréché *et al*. (9). Interestingly, the values stated on the manufacturer’s website are 100.0% and 98%, respectively (11). This discrepancy is likely attributable to the design of the cohort.

Previous accuracy evaluation studies are scarce and relatively old. Thus, the observed differences in performances can be attributed to variations in the manufacture of the reagents. The discrepancies in specificity observed between studies may also be due to the size of the population studied and the subjective selection of control cases. In addition, these parameters probably influence the proportion of equivocal results (13.3% in the N*Eh*D group in our study vs 9% in the study conducted by Boisseau (12) for the hemagglutination technique), which were considered negative in our study. Although we considered equivocal results to be negative, in practice, an equivocal result in a highly suggestive context would lead health care providers to consider an equivocal result as positive, or to repeat the test. Our research has also shown that optimizing the commercial threshold is crucial for enhancing the efficacy of commercial methods, but this requires access to a well-defined and well-conserved biobank.

It should be noted that the present study has limitations. Based on a multicenter biobank, the four techniques were performed in a single center. This could have artificially increased the performance of the hemagglutination technique, particularly. This technique is known to be associated with high interlaboratory variability due to environmental conditions, especially room temperature (13). Moreover, the availability of reagents should be considered, as this will impact the feasibility of adapting the current findings.

Another limitation is the study design. The very low prevalence of amoebic abscesses in high-income countries makes it impossible to carry out a non-retrospective study. Only studies conducted in an area with a high prevalence of amoebiasis could be prospective and allow for the predictive values of these tests to be calculated. From another point of view, the diversity of the sera included is a major strength of the study. Dysimmunitary diseases are increasingly common, and amoeba serology is part of systematic screening for donors of fecal matter in France (14), but not internationally (15). The occurrence of false-positive results in this population initially led to the rejection of donations. However, thanks to the optimization of the positive threshold through this study focused on ELISA methods, primarily used as an initial screening method, this issue should no longer be a concern. Furthermore, the inclusion of routine ELISA false-positive sera represents an intentional bias with a definite impact on the observed specificity. Another limitation is that the positive status of the serums for amebiasis was previously determined using different techniques. This may therefore call the gold standard into question.

Finally, this study also reveals that positive serology is very often associated with amoebic colitis (16, 17), although serology is not indicated for the diagnosis of this clinical form of amoebiasis (1, 18). The use of serological diagnosis for *Eh*A is therefore difficult to interpret in highly endemic areas, as it cannot distinguish between past and current infection or scarring amoebiasis (18–20). Even in our study outside an endemic area, we cannot rule out the possibility that some false positives were patients with an old infection, but with residual antibodies.

In conclusion, large comparative studies are needed to guide clinical microbiologists in their choices. This is even more important for parasitic diseases, such as amoebic abscesses, which are rare in routine practice. In low- to middle-income countries, reagent choice is also influenced by laboratory equipment and the availability of reagents. Our work provides baseline data for what would ideally be a prospective multicenter accuracy study.

## SUPPLEMENTAL MATERIAL

Supplemental tables and figure jcm.00179-25-s0001.docx Tables S1 to S7 and Figure S1

ASM does not own the copyrights to Supplemental Material that may be linked to, or accessed through, an article. The authors have granted ASM a non-exclusive, world-wide license to publish the Supplemental Material files. Please contact the corresponding author directly for reuse.
