# Supplementary material for: Serodiagnosis of amoebic abscess: a retrospective diagnostic accuracy study of kits marketed in Europe
Source: J Clin Microbiol. 2025 Oct 21;63(11):e00179-25. doi: 10.1128/jcm.00179-25 (PMC12607903; doi:10.1128/jcm.00179-25)
Supplement: Supplemental tables and figure — Tables S1 to S7 and Figure S1 [file jcm.00179-25-s0001.docx]

**Table S1**

**Inclusion criteria and number of serum samples for all categories**

| **CATEGORY** | **INCLUSION CRITERIA** |
| --- | --- |
| ***E. histolytica* abscesses (*Eh*A)** (n=79) | |
| Proved tissue amoebiasis (n=79) | Positive amoebic routine serology, consistent epidemiology, imaging and treatment with therapeutic success (other aetiologies excluded); and positive molecular testing if available |
| **Non-*E. histolytica* diseases (N*Eh*D)** (n=252) | |
| Parasitic diseases (n=134) | |
| Helminthiases (n=72) | |
| Echinococcosis (n=20) | Alveolar: imaging, positive serology (western blot) and diagnosis agreed at a multi-disciplinary meeting (other aetiologies excluded) (n=10)  Cystic: imaging, positive serology (western blot) and diagnosis agreed at a multi-disciplinary meeting (other aetiologies excluded) (n=10) |
| Strongyloidiasis (n=13) | Proved: positive serology and *Strongyloides* larvae in stools (positive direct examination and/or PCR) (n=11)  Probable: positive serology (n=2) |
| Toxocariasis (n=12) | Proved: positive serology (western blot), consistent clinical examination and treatment with therapeutic success (n=2)  Probable: positive serology (n=10) |
| Schistosomiasis (n=11) | *Schistosoma* eggs (positive direct examination) and/or positive PCR in stools, urine and/or biopsy; and positive serology (western blot) |
| Filariasis (n=9) | Proved: positive direct examination and/or positive PCR (n=8)  Probable: positive serology and consistent clinical examination (n=2) |
| Trichinosis (n=3) | Proved: positive serology (western blot), consistent clinical examination and treatment with therapeutic success (n=2)  Probable: positive serology (n=1) |
| Fasciolosis (n=3) | Proved: positive serology (western blot), consistent clinical examination and treatment with therapeutic success (n=2)  Probable: positive serology (n=1) |
| Cysticercosis (n=1) | Positive serology (western blot) consistent imaging and clinical examination |
| Protozoan diseases (n=62) | |
| Giardiasis (n=11) | *Giardia* spp*.* trophozoites/cysts in stools (positive direct examination and/or PCR) |
| Toxoplasmosis (n=11) | Recent: recent seroconversion with IgG and IgM (n=2)  Disseminated/cerebral/pulmonary/ocular: positive serology, symptoms, and positive PCR in blood/bronchoalveolar lavage/cerebrospinal fluid/aqueous humour (n=8)  Serological reactivation: IgG increase without symptoms (n=1) |
| Non-pathogenic amoeba carriage (n=10) | Presence of *Entamoeba coli* and/or *E. dispar* and/or *Dientamoeba fragilis* in stools (positive direct examination and/or DNA detection) |
| Cryptosporidiosis (n=10) | *Cryptosporidium* spp. oocysts in stools (positive direct examination and/or PCR) and digestive symptoms |
| Malaria (n=10) | Positive serology and undoubted malaria in medical history and/or recent diagnosis of malaria |
| Leishmaniasis (n=10) | Positive serology (western blot) and *Leishmania infantum* in blood and/or marrow (positive direct examination and/or PCR) |
| Other non-amoebic diseases (n=118) | |
| Immune dysfunction (n=60) | Presence of abnormal levels of rheumatoid factors and/or anti-nuclear antibodies and/or anti-citrullinated peptide antibodies (at least one parameter >2N) |
| Liver disease (n=29) | Patient with pyogenic abscess, solitary simple liver cyst, hepatocellular carcinoma, or hepatic metastases |
| Isolated positive serology (n=29) | Positive amoebic serology for one or more *E. histolytica* serology techniques for a patient whose acute episode may be clinically consistent with an amoebic abscess but whose diagnosis of amoebiasis has been rejected by a multi-disciplinary team and not treated; a serological trace of old amoebiasis cannot be completely ruled out. |
| **Healthy donors (HD)** (n=98) | |
| Healthy blood donors (n=80) | Blood donor serum samples supplied by the French Blood Establishment |
| Faecal transplant donors (n=18) | Serum sampled in faecal transplant screening context |
| ***E. histolytica* colitis (*Eh*C)** (n=13) (excluded from statistical analysis) | |
| Digestive amoebiasis (n=13) | *E. histolytica* in stools (positive DNA detection) |

**Table S2**

**Brief description of the four commercial kits analysed in this study**

| **Commercial kit** | **BORDIER**  ***E. histolytica* IgG ELISA** | **NOVATEC**  ***E. histolytica* IgG ELISA** | **ELI.H.A *Amoeba*^®^** | **ELITex Bicolor *Amoeba*^®^** |
| --- | --- | --- | --- | --- |
| Manufacturer | Bordier Affinity products SA (Crissier, Switzerland) | NovaTec Immunodiagnostica GmbH (Dietzenbach, Germany) | ELITech  Microbio^®^ (Signes, France) | ELITech  Microbio^®^ (Signes, France) |
| Method | ELISA | ELISA | Indirect haemagglutination | Latex agglutination |
| Antigen | Soluble antigens from *E. histolytica* trophozoites | *E. histolytica* antigens | Erythrocytes coated with a soluble endogenous *E. histolytica* antigen | Latex particles coated with total mixed antigen of E. *histolytica* |
| Sample type and volume | Serum: 5 µL | Serum: 10 µL | Serum: 25 µL | Serum: 25 µL |
| Handling time | 2h | 2h | 2.5h | 15min |
| Threshold | Negative: index<1 Positive: ≥1 | Negative: <11 NTU^a^ Positive: ≥11 NTU | Negative: titre <1:320^a^ Positive: titre ≥1:320 | Negative: no agglutination with 1:5 dilution  Positive: agglutination from 1:5 reaction dilution^b^ |
| Price (€)/test^c^ | 14.85 | 13.25 | 11.44 | 34.32 |
| Sample collection and preparation (manuals) | ‘ Store at 2-8°C if analyzed within a few days of collection,  otherwise store at -20°C or lower. Avoid freeze/thaw cycles.’ | ‘If the assay is performed within 5 days after sample collection, the samples should be kept at 2...8 °C; otherwise they should be aliquoted and stored deep-frozen (-70…-20 °C). If samples are stored frozen, mix thawed samples well before testing. Avoid repeated freezing and thawing.’ | ‘Use fresh serum or serum stored at -20°C, and not showing any sign of haemolysis, cloudiness or of contamination.  Avoid repeated freezing and thawing.’ | ‘Use freshly collected serum.  Serum samples can be stored for 24 hours at 2°- 8°C. If the test is not  not performed within 24 hours of collection, they should be frozen at -20°C. It is recommended to prepare aliquots to avoid successive freezing and thawing.’ |

^a^Equivocal results according to the manufacturer’s threshold were considered negative results for interpretation and analysis; ^b^Titre of the last positive dilution; ^c^If test is performed individually according to manufacturer’s recommendations (control, calibrators etc.); manufacturer’s catalogue VAT-free prices on 14 September 2023.

**Table S3**

**Demographic and diagnostic data for patients included in the biobank (except healthy blood donors).**

|  | **Number of samples** | **Male/female ratio** | **Median age**  (years) **(IQR)** | **Age range** (years) |
| --- | --- | --- | --- | --- |
| ***E. histolytica* abscesses (*Eh*A)** | **79** | **5.6** | **41 (22)** | **2-74** |
| Proved tissue amoebiasis | 79 | 5.6 | 41 (22) | 2-74 |
| **Non-*E. histolytica* diseases (N*Eh*D)** | **252** | **1.3** | **55 (33)** | **0.7-89** |
| **Parasitic diseases** | **134** | **1.5** | **43 (38)** | **0.7-87** |
| Helminthiases | 72 | 2.1 | 50 (43) | 4-85 |
| Echinococcosis | 20 | 1.2 | 61 (36) | 8-85 |
| Strongyloidiasis | 13 | 2.3 | 57 (43) | 4-77 |
| Toxocariasis | 12 | 2.0 | 64 (15) | 49-83 |
| Schistosomiasis | 11 | All males | 19 (16) | 9-19 |
| Filariasis | 9 | 2.0 | 29 (28) | 17-62 |
| Trichinosis | 3 | All males | 66 (28) | 40-68 |
| Fasciolosis | 3 | 0.5 | 26 (57) | 25-82 |
| Cysticercosis | 1 | Only female | 49 (NA) | 49 |
| Protozoan diseases | 62 | 1.1 | 38 (31) | 0.7-87 |
| Giardiasis | 11 | 4.5 | 34 (35) | 15-55 |
| Toxoplasmosis | 11 | 0.8 | 51 (26) | 25-68 |
| Non-pathogenic amoeba carriage | 10 | 1.5 | 36 (23) | 16-87 |
| Cryptosporidiosis | 10 | 0.7 | 34 (52) | 0.7-71 |
| Malaria | 10 | 0.3 | 30 (21) | 17-50 |
| Leishmaniasis | 10 | 1.5 | 40 (55) | 1-68 |
| **Other non-amoebic diseases** | **118** | **1.1** | **63 (21)** | **7-89** |
| Immune dysfunction | 60 | 0.5 | 64 (24) | 7-88 |
| Liver disease | 29 | 1.6 | 64 (11) | 12-84 |
| Isolated positive serology | 29 | 3.1 | 55 (31) | 9-89 |
| **Healthy donors (HD)** | ***98*** | ***Unavailable*** | ***Unavailable*** | ***Unavailable*** |
| Healthy blood donors | *80* | *Unavailable* | *Unavailable* | *Unavailable* |
| Faecal transplant donors | 18 | 1.3 | 25 (23) | 20-56 |
| ***E. histolytica* colitis (*Eh*C)** | **13** | **0.9** | **27 (27)** | **4-66** |
| Amoebic colitis | 13 | 0.9 | 27 (27) | 4-66 |
| **Total** | ***442* (362*)** | **1.6*** | **49* (32*)** | **0.7-89*** |

NA: not applicable

*Data calculated using the entire population, excluding healthy blood donors (demographic data unavailable)

**Figure S4**

**Representation of titre proportions for the two semi-quantitative techniques, according to the group**

Serum sample titre proportions assessed using ELITex Bicolor *Amoeba*^®^ (**A-B**) and ELI.H.A *Amoeba*^®^ (**C-D**), according to the *Eh*A group (n=79) (**A, C**), the non *Eh*A group (n=350) (**B, D**), or detailed by subgroup (**E**) using the ELI.H.A *Amoeba*^®^ method.

*Eh*A: *E. histolytica* abscess (n=79); non *Eh*A group: healthy donors (HD) and non-*E. histolytica* diseases (N*Eh*D)(n=350); HEL: helminthiases (=72); PRO: protozoan diseases (n=62); DYS: immune dysfunctions (n=60); ONAD: other non-amoebic diseases (liver diseases and isolated positive serology) (n=58); HBD: healthy blood donors (n=80); FTD: faecal transplant donors (n=18).


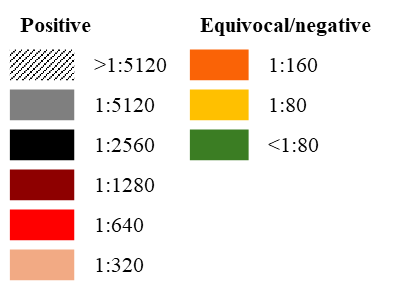


|  |  | **Bordier ELISA** | | **NovaTec ELISA** | | **ELI.H.A *Amoeba*^®^** | | **ELITex Bicolor *Amoeba*^®^** | |
| --- | --- | --- | --- | --- | --- | --- | --- | --- | --- |
| **CATEGORY** | **No.** | **Total FP** | **Isolated FP** | **Total FP** | **Isolated FP** | **Total FP** | **Isolated FP** | **Total FP** | **Isolated FP** |
| **Non-*E. histolytica* diseases (N*Eh*D)** | **252** | **49 (19.4)** | **22 (8.7)** | **72 (28.6)** | **47 (18.9)** | **7 (2.8)** | **3 (1.2)** | **5 (2.0)** | **3 (1.2)** |
| **Parasitic diseases** | **134** | **26 (19.4)** | **10 (13.9)** | **35 (23.9)** | **21 (15.7)** | **2 (1.5)** | **1 (0.7)** | **4 (3.0)** | **2 (1.5)** |
| **Helminthiases** | **72** | **14 (19.4)** | **7 (9.7)** | **20 (27.8)** | **13 (18.1)** | **0** | **0** | **2 (2.8)** | **2 (2.8)** |
| Echinococcosis | 20 | 5 (25.0) | 2 (10.0) | 10 (50.0) | 7 (35.0) | 0 | 0 | 1 (5.0) | 1 (5.0) |
| Strongyloidiasis | 13 | 3 (23.1) | 0 | 5 (38.5) | 2 (15.4) | 0 | 0 | 0 | 0 |
| Toxocariasis | 12 | 1 (8.3) | 1 (8.3) | 0 | 0 | 0 | 0 | 0 | 0 |
| Schistosomiasis | 11 | 2 (18.2) | 2 (18.2) | 1 (9.1) | 1 (9.1) | 0 | 0 | 0 | 0 |
| Filariasis | 9 | 3 (33.3) | 2 (22.2) | 4 (44.4) | 3 (33.3) | 0 | 0 | 1 (11.1) | 1 (11.1) |
| Trichinosis | 3 | 0 | 0 | 0 | 0 | 0 | 0 | 0 | 0 |
| Fasciolosis | 3 | 0 | 0 | 0 | 0 | 0 | 0 | 0 | 0 |
| Cysticercosis | 1 | 0 | 0 | 0 | 0 | 0 | 0 | 0 | 0 |
| **Protozoan diseases** | **62** | **12 (19.4)** | **3 (4.8)** | **16 (25.8)** | **8 (12.9)** | **2 (3.2)** | **1 (1.6)** | **2 (3.2)** | **0** |
| Giardiasis | 11 | 2 (18.2) | 0 | 4 (36.4) | 2 (18.2) | 0 | 0 | 0 | 0 |
| Toxoplasmosis | 11 | 0 | 0 | 2 (18.2) | 2 (18.2) | 0 | 0 | 0 | 0 |
| Non-pathogenic amoeba carriage | 10 | 2 (20.0) | 1 (10.0) | 2 (20.0) | 1 (10.0) | 1 (10.0) | 0 | 1 (10.0) | 0 |
| Cryptosporidiosis | 10 | 1 (10.0) | 0 | 0 | 0 | 0 | 0 | 1 (10.0) | 0 |
| Malaria | 10 | 2 (20.0) | 1 (10.0) | 2 (20.0) | 1 (10.0) | 1 (10.0) | 1 (10.0) | 0 | 0 |
| Leishmaniasis | 10 | 5 (50.0) | 1 (10.0) | 6 (60.0) | 2 (20.0) | 0 | 0 | 0 | 0 |
| **Other non-amoebic diseases** | **118** | **23 (19.5)** | **12 (10.2)** | **36 (30.5)** | **26 (22.0)** | **5 (4.2)** | **2 (1.7)** | **1 (0.8)** | **1 (0.8)** |
| Immune dysfunction | 60 | 5 (8.3) | 3 (5.0) | 13 (21.7) | 11 (18.3) | 2 (3.3) | 2 (3.3) | 0 | 0 |
| Liver disease | 29 | 4 (13.8) | 1 (3.4) | 13 (44.8) | 10 (34.5) | 0 | 0 | 0 | 0 |
| Isolated positive serology | 29 | 14 (48.3) | 8 (27.6) | 10 (34.5) | 5 (17.2) | 3 (10.3) | 0 (0.0) | 1 (3.4) | 1 (3.4) |
| **Healthy donors (HD)** | **98** | **5 (5.1)** | **4 (4.1)** | **4 (4.1)** | **3 (3.1)** | **0** | **0** | **0** | **0** |
| Healthy blood donors | 80 | 5 (6.3) | 4 (5.0) | 4 (5.0) | 3 (3.8) | 0 | 0 | 0 | 0 |
| Faecal transplant donors | 18 | 0 | 0 | 0 | 0 | 0 | 0 | 0 | 0 |
| **Total** | 350 | 54 (15.4) | 26 (7.4) | 76 (21.7) | 50 (14.3) | 7 (2.0) | 3 (0.9) | 5 (1.4) | 3 (0.9) |

N*Eh*D: non-*E. histolytica* diseases; HD: healthy donors; FP: false positive, n(% in the group/subgroup).
Isolated FP: FP observed only with the mentioned technique; total FP: isolated FP of the mentioned technique and FP also observed with one or more techniques.

**Table S5**

**Categorisation of false-positive results with at least two of the four studied amoebic serology techniques or only one technique**

**Table S6**

**Performances of semi-quantitative techniques based on consideration of equivocal results**

|  | No. tested | NovaTec ELISA | | | ELI.H.A *Amoeba*^®^ | | |
| --- | --- | --- | --- | --- | --- | --- | --- |
|  |  | Pos | Equivocal | Neg | Pos | Equivocal | Neg |
| *Eh*A group  (disease) | 79 | 69 | 4 | 6 | 75 | 2 | 2 |
| N*Eh*D + HD groups  (no disease) | 350 | 76 | 90 | 184 | 7 | 46 | 297 |
| Se_1_ (%) | 429 | 87.3  [87.3-88.6] | | | 94.9  [94.9-96.2] | | |
| Se_2_ (%) |  | 92.4  [92.4-993.7] | | | 97.5  [97.4-98.8] | | |
| Sp_1_ (%) |  | 78.3  [78.3-79.5] | | | 98.0  [98.0-99.3] | | |
| Sp_2_ (%) |  | 52.6  [52.6-53.7] | | | 84.9  [84.8-86.1] | | |
| AUC_1_ |  | 0.905  [0.857-0.953] | | | 0.983  [0.963-1.003] | | |
| AUC_2_ |  | 0.725  [0.682-0.762] | | | 0.912  [0.885-0.935] | | |
| LR^+^_1_ |  | 4.02  [4.02-4.05] | | | 47.47  [47.38-47.61] | | |
| LR^+^_2_ |  | 1.95  [1.05-1.96] | | | 6.44  [6.43-6.47] | | |
| LR^-^_1_ |  | 0.16  [0.16-0.17] | | | 0.05  [0.05-0.06] | | |
| LR^-^_2_ |  | 0.14  [0.14-0.15] | | | 0.03  [0.03-0.04] | | |
| Accuracy_1_ |  | 80.0  [79.9-81.2] | | | 97.4  [97.4-98.7] | | |
| Accuracy_2_ |  | 59.9  [59.9-61.1] | | | 87.2  [83.9-90.5] | | |

Pos: positive; Neg: negative; *Eh*A: *E. histolytica* abscesses; N*Eh*D: non-*E. histolytica* diseases; HD: healthy donors; Se: sensitivity; Sp: specificity; AUC: area under the curve; LR^+^: positive likelihood ratio, LR^-^: negative likelihood ratio; X_1_ : equivocal considered as negative, X_2_ : equivocal considered as positive,

**Table S7**

**Serum data for patients with *E. histolytica* colitis (*Eh*C group) (n=13)**

| **No.** | BORDIER  *E. histolytica* IgG ELISA | | NOVATEC  *E. histolytica* IgG ELISA | | ELI.H.A *Amoeba*^®^ | | ELITex Bicolor *Amoeba*^®^ | |
| --- | --- | --- | --- | --- | --- | --- | --- | --- |
|  | Index | Result | Index (NTU) | Result | Titre | Result | Titre | Result |
| **1** | 2.30 | **pos** | 16.73 | **pos** | 2 560 | **pos** | 20 | **pos** |
| **2** | 1.82 | **pos** | 12.759 | **pos** | 5 120 | **pos** | <5 | neg |
| **3** | 4.57 | **pos** | 26 | **pos** | 5 120 | **pos** | >40 | **pos** |
| **4** | 0.28 | neg | 3.927 | neg | <80 | neg | <5 | neg |
| **5** | 4.38 | **pos** | 26.098 | **pos** | 5 120 | **pos** | >40 | **pos** |
| **6** | 3.43 | **pos** | 20.683 | **pos** | >5 120 | **pos** | >40 | **pos** |
| **7** | 3.676 | **pos** | 23.341 | **pos** | 5 120 | **pos** | 20 | **pos** |
| **8** | 0.457 | neg | 6.110 | neg | <80 | neg | <5 | neg |
| **9** | 1.801 | **pos** | 14.524 | **pos** | 2 560 | **pos** | 10 | **pos** |
| **10** | 3.921 | **pos** | 21.476 | **pos** | >5 120 | **pos** | >40 | **pos** |
| **11** | 0.808 | neg | 8.622 | neg | <80 | neg | <5 | neg |
| **12** | 0.862 | neg | 6.479 | neg | 320 | **pos** | 10 | **pos** |
| **13** | 2.058 | **pos** | 25.862 | **pos** | >5 120 | **pos** | 20 | **pos** |

pos: positive; neg: negative
